# Supplementary material for: One-Step Reverse-Transcription Digital PCR for Reliable Quantification of Different Pepino Mosaic Virus Genotypes
Source: Plants (Basel). 2020 Mar 4;9(3):326. doi: 10.3390/plants9030326 (PMC7154864; doi:10.3390/plants9030326)
Supplement: Supplementary file 1 [file plants-09-00326-s001.pdf]

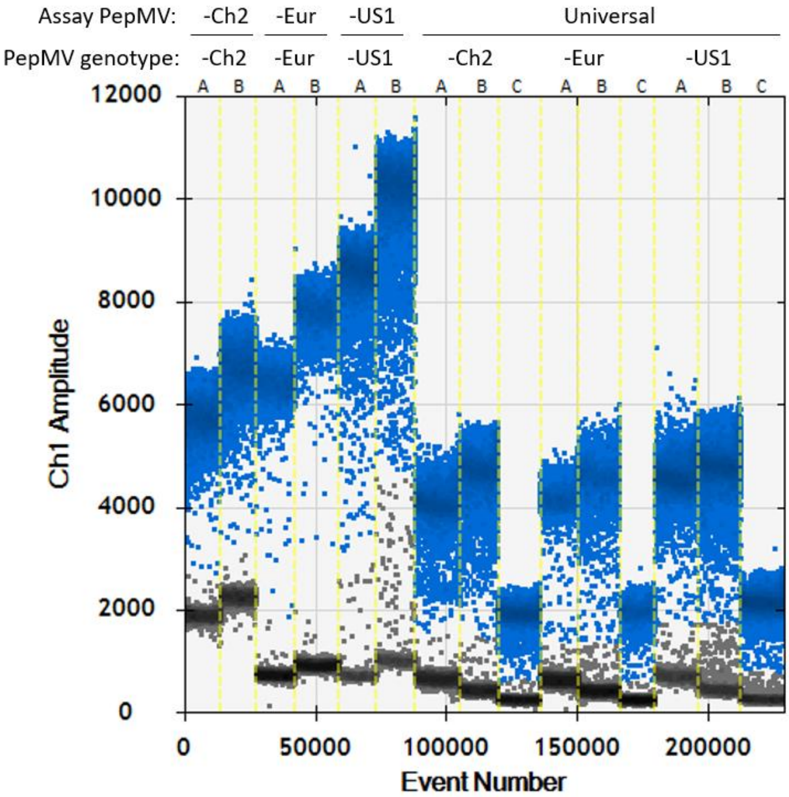

Figure S1. Influence of the concentrations of the primers and probe on the separation of the positive and negative droplets for the QX100/200 platform. Samples containing PepMV-Ch2, PepMV-Eur and PepMV-US1 were analysed using different final concentrations of primers and probe: (A) 900 nM primers and 200 nM probe for the specific PepMV-Ch2, PepMV-Eur and PepMV-US1 assays, and 200 nM primers and 400 nM probe for the PepMV-universal assay; (B) 900 nM primers and 250 nM probe for the specific PepMV-Ch2, PepMV-Eur and PepMV-US1 assays, and 450 nM primers and 250 nM probe for the PepMV-universal assay; and (C) 300 nM primers and 100 nM probe for the PepMV-universal assay. Based on the resolution, the optimal primer and probe concentrations were the same as those used for the RT-qPCR; 900 nM primers and 200 nM probe for the specific PepMV-Ch2, PepMV-Eur and PepMV-US1 assays, and 200 nM primers and 400 nM probe for the PepMV-universal assay.

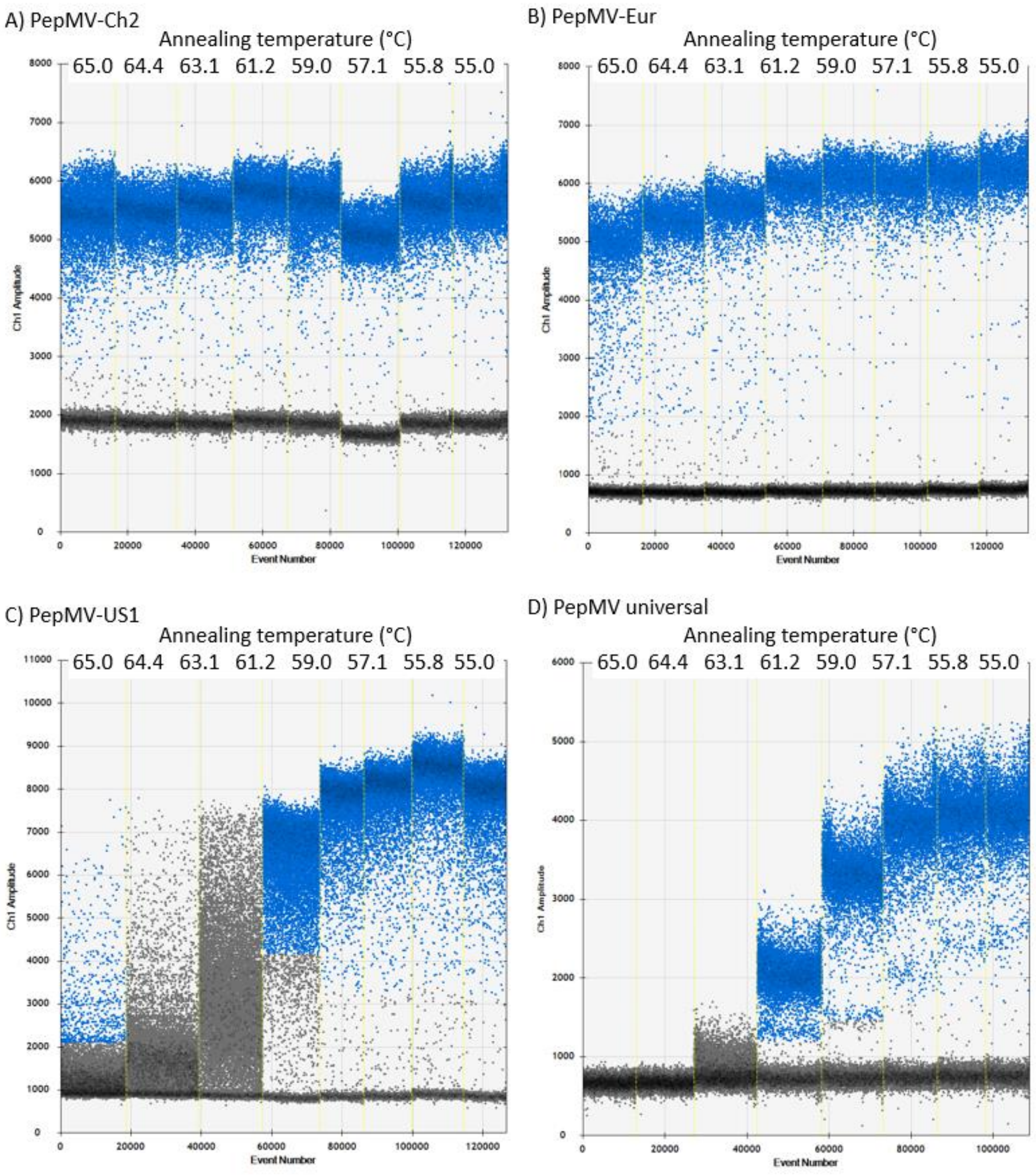

Figure S2. Influence of the annealing temperatures on separation of the positive and negative clusters on the QX100/200 platform. The PepMV-Ch2, PepMV-Eur and PepMV-US1 samples were analysed using the PepMV-genotype-specific (A, PepMV-Ch2; B, PepMV-Eur; C, PepMV-US1) and PepMV-universal (D) assays, and eight annealing temperatures in the range of 55 °C to 65 °C. For the PepMV-universal assay, the PepMV-Ch2 sample was tested. The optimal annealing temperature for all of the assays was ~56 °C.

Table S1. RT-qPCR results and selected parameters from the RT-dPCR analysis with serial dilutions of PepMV RNA extracted from infected leaf material of the test plants. The dilution series (10-fold) of PepMV-positive RNA samples were prepared with RNAse-free water; and for PepMV-Ch2, also in RNA isolated from leaf material of healthy test plants.

Sample	RNA dilution	RT-qPCR Cq	QX100/200[b] (for 20 µL reaction mixture)				Naica[b] (for 25 µL reaction mixture)			
			Positive droplets (n)	Droplets analysed (n)	λ[b]	P[c]	Positive droplets (n)	Droplets analysed (n)	λ[b]	P[c]
PepMV-Ch2	10^{-3}	21.1	15392	15395	8.5432	0.9998	20189	20336	4.9297	0.9928
		21.1	18000	18002	9.1051	0.9999	16942	17044	5.1186	0.9940
		21.0	16341	16341	NA[d]	1.0000[d]	19856	20025	4.7748	0.9916
	10^{-4}	24.4	10599	18216	0.8719	0.5819	10543	27627	0.4807	0.3816
		24.5	8929	15570	0.8521	0.5735	11971	27922	0.5599	0.4287
		24.5	9737	17112	0.8417	0.5690	9735	24500	0.5064	0.3973
	10^{-5}	28.1	1339	16497	0.0847	0.0812	1153	23713	0.0498	0.0486
		28.3	1398	16654	0.0877	0.0839	1355	25737	0.0541	0.0526
		28.3	1267	15926	0.0829	0.0796	797	17700	0.0461	0.0450
	10^{-6}	31.4	127	16285	0.0078	0.0078	109	28868	0.0038	0.0038
		31.6	118	15582	0.0076	0.0076	130	30991	0.0042	0.0042
		31.5	103	14703	0.0070	0.0070	100	26203	0.0038	0.0038
	10^{-7}	35.0	17	15724	0.0011	0.0011	6	23680	0.0003	0.0003
		34.6	14	15273	0.0009	0.0009	19	28675	0.0007	0.0007
		36.5	19	15784	0.0012	0.0012	12	21405	0.0006	0.0006
	10^{-8}	Negative	1	15801	0.0001	0.0001	4	30496	0.0001	0.0001
		Negative	2	15581	0.0001	0.0001	64[e]	31536	0.0020	0.0020
		37.3	2	17456	0.0001	0.0001	0	21514	0.0000	0.0000
PepMV-Eur	10^{-3}	22.5	13213	13869	3.0513	0.9527	15575	20336	1.4519	0.7659
		22.4	10168	10782	2.8656	0.9431	13367	17044	1.5337	0.7843
		22.3	14470	15328	2.8828	0.9440	15597	20025	1.5090	0.7789
	10^{-4}	25.6	3637	14236	0.2950	0.2555	3770	27627	0.1467	0.1365
		25.6	3271	13105	0.2871	0.2496	4329	27922	0.1685	0.1550
		25.5	4126	16325	0.2913	0.2527	3676	24500	0.1626	0.1500
	10^{-5}	29.4	433	15406	0.0285	0.0281	362	23713	0.0154	0.0153
		28.8	349	11244	0.0315	0.0310	442	25737	0.0173	0.0172
		28.9	464	16171	0.0291	0.0287	278	17700	0.0158	0.0157
	10^{-6}	38.2	30	14882	0.0020	0.0020	44	28868	0.0015	0.0015

		38.3	36	11367	0.0032	0.0032	38	30991	0.0012	0.0012
		39.7	36	17081	0.0021	0.0021	25	26203	0.0010	0.0010
	10^{-7}	Negative	1	15512	0.0001	0.0001	0	23680	0.0000	0.0000
		Negative	2	10805	0.0002	0.0002	2	28675	0.0001	0.0001
		Negative	7	16641	0.0004	0.0004	2	21405	0.0001	0.0001
	10^{-8}	Negative	0	9404	0.0000	0.0000	0	30496	0.0000	0.0000
		Negative	0	9131	0.0000	0.0000	1	31536	0.0000	0.0000
		Negative	1	17031	0.0001	0.0001	1	21514	0.0000	0.0000
PepMV-US1	10^{-3}	20.7	12896	12896	NA[d]	1.0000[d]	21525	21528	8.8785	0.9999
		20.4	14169	14170	9.5589	0.9999[d]	178	178[d]	NA[d]	1.0000[d]
		20.5	15130	15130	NA[d]	1.0000[d]	25579	25602	7.0149	0.9991
	10^{-4}	23.7	12082	13860	2.0535	0.8717	18071	25859	1.2001	0.6988
		23.9	11802	13382	2.1365	0.8819	18631	26293	1.2330	0.7086
		23.8	9685	10916	2.1824	0.8872	4747	6856[d]	1.1789	0.6924
	10^{-5}	27.6	2503	13702	0.2017	0.1827	2301	21811	0.1115	0.1055
		27.8	2333	12178	0.2127	0.1916	2536	24417	0.1097	0.1039
		27.4	1967	10613	0.2050	0.1853	2511	23632	0.1123	0.1063
	10^{-6}	30.9	244	14633	0.0168	0.0167	260	27450	0.0095	0.0095
		31.5	221	13475	0.0165	0.0164	235	26730	0.0088	0.0088
		31.0	158	10745	0.0148	0.0147	185	19513	0.0095	0.0095
	10^{-7}	34.8	20	12796	0.0016	0.0016	22	23095	0.0010	0.0010
		35.8	21	13109	0.0016	0.0016	22	25366	0.0009	0.0009
		34.7	25	12015	0.0021	0.0021	18	27614	0.0007	0.0007
	10^{-8}	Negative	1	15425	0.0001	0.0001	0	95[d]	0.0000	0.0000
		Negative	0	14150	0.0000	0.0000	1	27073	0.0000	0.0000
		Negative	1	10825	0.0001	0.0001	3	28461	0.0001	0.0001
Mix[a]	10^{-3}	20.6	13652	13854	4.2281	0.9854	21528	21528	NA[d]	1.0000[d]
		20.4	11336	11507	4.2090	0.9851	178	178[d]	NA[d]	1.0000[d]
		20.6	14838	15007	4.4864	0.9887	25563	25602	6.4869	0.9985
	10^{-4}	23.9	9196	10623	2.0074	0.8657	20452	25859	1.5650	0.7909
		23.8	11043	12401	2.2118	0.8905	21121	26293	1.6260	0.8033
		23.9	14685	16513	2.2009	0.8893	5336	6856[d]	1.5064	0.7783
	10^{-5}	27.5	3408	15876	0.2416	0.2147	3048	21811	0.1505	0.1397
		27.8	3621	16918	0.2408	0.2140	3384	24417	0.1492	0.1386
		27.2	3187	14889	0.2409	0.2141	3340	23632	0.1524	0.1413

	10^{-6}	31.1	243	13873	0.0177	0.0175	314	27450	0.0115	0.0114
		31.9	286	15205	0.0190	0.0188	287	26730	0.0108	0.0107
		31.8	306	16155	0.0191	0.0189	222	19513	0.0114	0.0114
	10^{-7}	34.6	28	15668	0.0018	0.0018	28	23095	0.0012	0.0012
		34.5	22	13611	0.0016	0.0016	24	25366	0.0009	0.0009
		35.4	20	11863	0.0017	0.0017	19	27614	0.0007	0.0007
	10^{-8}	38.7	1	13494	0.0001	0.0001	0	95[d]	0.0000	0.0000
		Negative	1	13384	0.0001	0.0001	6	27073	0.0002	0.0002
		Negative	0	9358	0.0000	0.0000	8	28461	0.0003	0.0003
PepMV-Ch2 (in RNA from healthy plants)	10^{-4}	25.0	5671	10327	0.7966	0.5491	10676	26713	0.5103	0.3997
		25.0	4915	9321	0.7493	0.5273	9697	24894	0.4935	0.3895
		25.1	6486	11740	0.8040	0.5525	9021	23019	0.4974	0.3919
	10^{-5}	28.4	900	12129	0.0771	0.0742	1369	28906	0.0485	0.0474
		28.5	905	12070	0.0779	0.0750	1336	27431	0.0499	0.0487
		28.3	1023	13461	0.0790	0.0760	30	470[d]	0.0660	0.0638
	10^{-6}	31.8	73	10467	0.0070	0.0070	147	30253	0.0049	0.0049
		31.6	105	12168	0.0087	0.0086	127	27407	0.0046	0.0046
		31.9	88	12786	0.0069	0.0069	143	29076	0.0049	0.0049
	10^{-7}	35.2	11	10505	0.0010	0.0010	4	4873[d]	0.0008	0.0008
		34.4	9	10853	0.0008	0.0008	17	25268	0.0007	0.0007
		34.8	9	14024	0.0006	0.0006	17	28488	0.0006	0.0006
	10^{-8}	37.4	0	8183[d]	0.0000	0.0000	4	28989	0.0001	0.0001
		38.3	0	10533	0.0000	0.0000	1	25611	0.0000	0.0000
		Negative	0	14036	0.0000	0.0000	1	29418	0.0000	0.0000
	10^{-9}	Negative	1	5187[d]	0.0002	0.0002	1	29490	0.0000	0.0000
		Negative	1	9438	0.0001	0.0001	0	27432	0.0000	0.0000
		Negative	0	15445	0.0000	0.0000	2	29169	0.0001	0.0001

Cq, quantification cycle; [a] mix of different PepMV genotypes tested with PepMV-universal assay; [b] mean number of copies per droplet = -ln (1-(number of positive droplets) / (number of accepted droplets)) [1]. The concentrations of PepMV RNA copy numbers per μL RNA sample were calculated through division of λ by the RNA volume per droplet (RNA volume per droplet = (droplet volume) / (total reaction volume) × (RNA sample volume); the droplet volumes assigned by the manufacturer were used: QX100/200, 0.85 nL; Naica, 0.43 nL); [c] fraction of positive droplets (probability that droplet is full) = (number of positive droplets) / (number of accepted droplets); [d] data were excluded from subsequent analysis if there were <9.000/ <15.000 droplets per 20 μL/ 25 μL reaction, or if >99.99% of the droplets were positive (NA, not applicable: most of the droplets that were analysed contained target copies, and so application of Poisson's law was not possible); [e] excluded from further analysis because pipetting error was assumed.

References

1. Huggett, J.F.; Foy, C.A.; Benes, V.; Emslie, K.; Garson, J.A.; Haynes, R.; Hellemans, J.; Kubista, M.; Mueller, R.D.; Nolan, T.; et al. The digital MIQE guidelines: Minimum information for publication of quantitative digital PCR experiments. *Clin. Chemist*. **2013**, *59*, 892–902, doi:10.1373/clinchem.2013.206375.

© 2020 by the authors. Submitted for possible open access publication under the terms and conditions of the Creative Commons Attribution (CC BY) license (http://creativecommons.org/licenses/by/4.0/).
